# A clinical perspective on surgical and diagnostic strategies for neonatal partial shone complex: Insights from a case report

**DOI:** 10.1016/j.ijscr.2024.110754

**Published:** 2024-12-17

**Authors:** Ayham Qatza, Moumina Baroudi, Abdullah Dukhan, Ahmed Sheikh Sobeh, Nabeha Alibrahim, Saleh Takkem

**Affiliations:** a Faculty of Medicine, Hama University, Hama, Syria; b Resident Cardiologist, Department of Cardiology, Hama National Hospital, Hama, Syria; c Department of Dermatology, Hama National Hospital, Hama, Syria; d The Internist Cardiologist, Department of Cardiology, Al Watani Hospital, Hama, Syria

**Keywords:** Shone complex, Parachute mitral valve, Mitral stenosis, Aortic stenosis, Coarctation of the aorta

## Abstract

**Introduction and clinical importance:**

Shone complex (SC) is a rare multilevel congenital heart disease (CHD) characterized by four left-sided heart obstructive lesions: parachute mitral valve, supravalvular mitral ring, subaortic stenosis, and coarctation of the aorta (CoA), accounting for 0.6–0.7 % of CHD cases.

**Case presentation:**

A 4-week-old male neonate presented with severe respiratory distress, tachycardia (150 beats/min), tachypnea (40/min), and hypoxia (80 % saturation). Blood pressure was 90/55 mmHg in the upper arms; lower extremity measurements were challenging. ECG showed a heart rate of 150 beats/min, normal sinus rhythm, left axis deviation, and left ventricular (LV) hypertrophy. Transthoracic echocardiography revealed mild concentric LV hypertrophy and reduced ejection fraction (45 %). A supramitral ring led to severe supravalvular mitral stenosis, and a bicuspid aortic valve caused moderate aortic stenosis. Suprasternal views confirmed severe CoA distal to the left subclavian artery. The patient underwent successful CoA repair at five months, with ongoing surveillance for other defects. One year later, he remained stable with no significant pressure gradient changes.

**Clinical discussion:**

SC presents significant clinical challenge due to its associated congenital anomalies. Early echocardiographic diagnosis and timely surgical intervention are essential for optimizing patient outcomes, given the variability in severity and the potential for complications. Multidisciplinary management is crucial for addressing the complexities of this condition.

**Conclusion:**

This case illustrates effective staged surgical management of partial SC, emphasizing early diagnosis and the utility of point-of-care ultrasound.

## Highlights

• The shone complex includes multiple left heart obstructive lesions, impacting blood flow and cardiac function.
• Early identification through echocardiography improves management and patient outcomes significantly.
• Staged surgeries address structural abnormalities; a multidisciplinary care improves treatment efficacy and holistic care.
• Patient education promotes understanding of the condition and adherence to treatment plans.
• Genetic counselling can provide insights for families with hereditary patterns of the shone complex.

## 1 Introduction

Shone complex (SC) is a rare multilevel congenital heart disease (CHD) involving four levels of obstructive lesions of the left-side of the heart: beginning with a supravalvular mitral ring, followed by parachute mitral valve (PMV), subaortic stenosis, and coarctation of the aorta (CoA) [1]. When SC involves two or three levels of obstructive lesions, it is called a non-typical or partial SC, which is the most common form of cases [2]. It accounts for 0.6–0.7 % of all CHD [3]. Most patients are diagnosed in childhood, but some cases have been rarely reported in adults in medical literature [1]. Echocardiography may diagnose or suggest the presence of SC, while multimodal imaging provides a more comprehensive evaluation of intra- and extra-cardiac obstructive lesions [4]. Childhood management usually requires cardiac surgery, commonly involving surgical coarctation repair, subaortic resection, and mitral valve repair [5]. This paper describes a 4-week-old male neonate diagnosed with partial SC. Following a successful surgical procedure, the patient's condition stabilized. By reporting this case, we aim to explain the pathophysiology of this syndrome, diagnostic approaches, its prognosis, and the different therapeutic strategies suggested, hoping to raise additional awareness of this rare case.

## 2 Case presentation

**Fig. 1:**
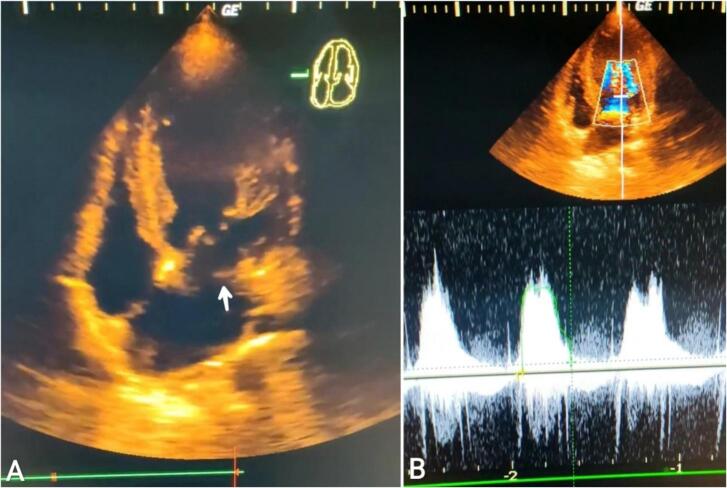
Two-dimensional (2D) transthoracic echocardiography: Image (A): Apical 4-chamber view showing membranous supramitral ring (white arrow). Image (B): Continuous wave doppler across the mitral valve demonstrates severe supravalvular mitral stenosis with a mean diastolic pressure gradient of 12 mmHg.

**Fig. 2:**
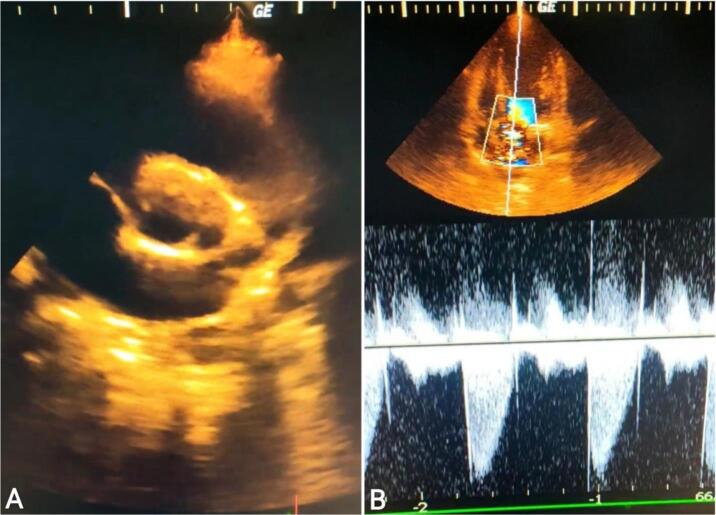
Image (A): Transthoracic short-axis view displays a bicuspid aortic valve. Image (B): Continuous wave doppler across the aortic valve shows oderate aortic stenosis with Vmax at 3.5 m/s.

**Fig. 3:**
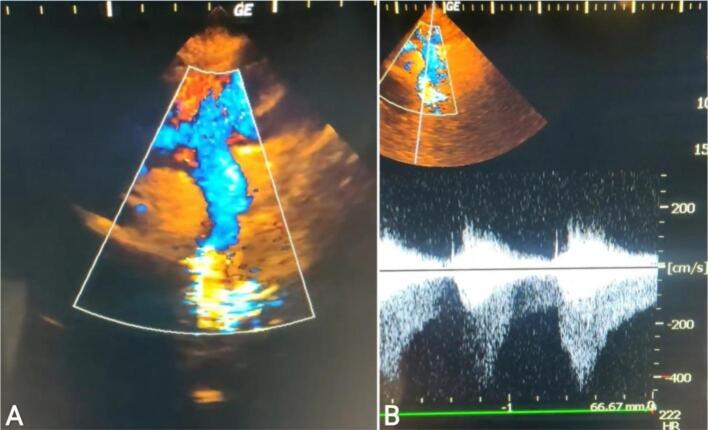
Image (A): A suprasternal view of the aorta reveals turbulent blood flow in the discending aorta, consistent with CoA. Image (B): Continuous wave doppler across the coarctation segment, presenting a peak velocity of 4 m/s and an extended speed reduction during diastole (diastolic tail).

**Fig. 4:**
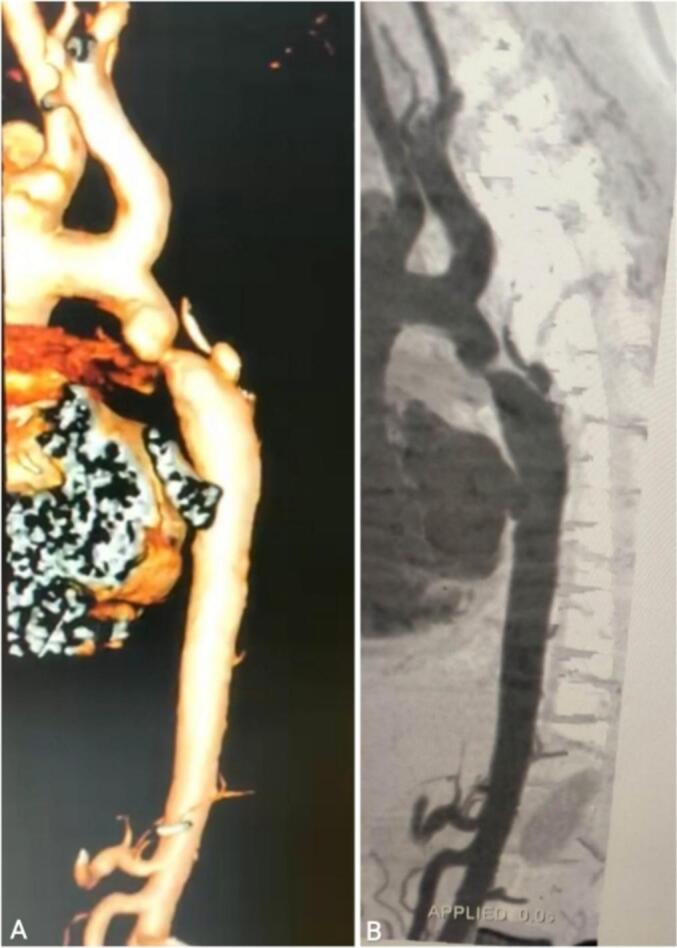
Two-dimensional (B) and three-dimensional (A) computed tomography scan imaging of coarctation of the aorta showing the discrete narrowing site at the aortic isthmus level.

A 4-week-old male neonate presented to the emergency department with severe respiratory distress. The pediatric physician observed a cardiac murmur and referred the patient to our specialist clinic. The patient's medical history was not known, and he was born at 39 weeks by vaginal delivery with routine prenatal care. The mother did not observe any unusual findings during the whole pregnancy, and she denied the use of tobacco, alcohol, and drugs. His initial vital signs on arrival showed a heart rate of 150 beats per minute (beats/min), a respiratory rate of 40/min, and hypoxia at an oxygen saturation of 80 % on room air. Blood pressures of the upper arms were 90/55 mm of mercury (mm Hg), while it was difficult to obtain accurate measurements in the lower extremities. The patient looked unwell and cyanotic, but the general examination was unremarkable. Cardiac auscultation revealed a grade III holosystolic murmur that was heard overall in the precordium. Laboratory blood tests were within normal range. The electrocardiogram (ECG) depicted a heart rate of 150 beats/min, normal sinus rhythm, left axis deviation, and signs of left ventricular (LV) hypertrophy. Given the patient's exhaustion, he had been transferred to the neonatal intensive care unit and placed on a high-flow nasal cannula. Two-dimensional (2D) transthoracic echocardiography (TTE) revealed mild concentric LV hypertrophy with a mildly reduced ejection fraction (EF) of 45 %. Apical four-chamber view showed a morphologically normal mitral valve (MV), but a supramitral ring was noted, resulting in severe supravalvular mitral stenosis with a mean transmembrane pressure gradient (P-mean) of 12 mmHg [Fig. 1]. There was also mild left atrial dilation. A bicuspid aortic valve was recognized which caused a mederate aortic stenosis. In addition, the maximum jet velocity (Vmax) of 3.5 m per second and a mean pressure gradient of 34 mmHg [Fig. 2]. Suprasternal views were obtained, which demonstrated severe CoA (diameter of approximately 2 mm) distal to the left subclavian artery origin [Fig. 3]. Measurements of the ascending aorta were 7–8 mm before the narrowing segment and 5 mm after it. Doppler assessment of the aorta displayed a maximal velocity of 4 m/s across the stenotic region and a maximal pressure gradient of 64 mmHg with the presence of a shark tooth pattern. A continuous diastolic flow pattern was noted in the abdominal aorta (indicating CoA) [Fig. 3]. Other abnormalities were mild regurgitation of the tricuspid valve with a maximal velocity of 2.5 m/s and mild elevation in pulmonary arterial systolic pressure (PASP) of 30–35 mmHg. Further investigations, including multi-detector computed tomographic (MDCT) angiography, clearly delineated the presence of severe CoA [Fig. 4]. Based upon these findings, a CHD was established, and a partial form of SH was the final diagnosis. The surgical intervention for the neonate was postponed until the fifth month due to the family's financial constraints, necessitating a conservative management approach with periodic observation instead of immediate surgery. In the fifth month, the patient underwent surgical repair for the CoA, and successful outcomes were achieved. Cardiology consultants decided to relieve management of other defects with an annual evaluation by echocardiography. During the one-year follow-up, the patient seemed to be in a good state, and there weren't significant differences in pressure gradients from those previously measured.

## 3 Discussion

SC was first described by John Shone in 1963 [6]. Its complete form consists of a supra-mitral valve ring, subaortic stenosis, PMV, and CoA. Otherwise, the incomplete form is more common and includes a left ventricular inlet defect (PMV, mitral stenosis congenitally or supravalvular mitral ring) along with at least one LV escape lesion (bicuspid aortic valve, subvalvular aortic stenosis, CoA) [7]. Thus, the presence of these defects should prompt searching for other cardiac abnormalities. SC typically manifests early, with a median diagnosis age of 14 days in a retrospective study. Another cohort study reported a prevalence of 0.7 % in 4000 CHD cases, highlighting that the incomplete form is more common than the complete [4]. It is proposed that the pathogenesis of SC originates from a congenital MV anomaly affecting the LV inflow tract during embryogenesis [5]. Subsequent underdevelopment of the LV results in various degrees of left ventricular outflow tract (LVOT) obstruction and aortic coarctation. PMV is characterized by the convergence of the chordae-tendinae from both MV leaflets onto a single papillary muscle, resulting in restricted valve opening, subvalvular obstruction, and regurgitation. The supravalvular mitral ring, a membrane-like peripheral ridge originating from the left atrial wall overlying the MV, can attach to the MV. Subsequent impairment of the leaflets opening causes MV inflow obstruction. This ring, if large enough, may protrude into the MV inflow track, exacerbating obstruction. In addition, turbulent flow can lead to a progressive increase in the thickness of the supravalvular membrane or ridge, aggravating mitral inflow obstruction [8]. Furthermore, Shone et al. observed that MV obstruction is the most significant lesion. Those with the most severe mitral obstruction presented considerably elevated pulmonary artery pressures and had the worst prognostic outcomes [2]. CoA, a congenital narrowing of the aorta, is classified into 3 types: preductal, ductal, or postductal; named depending on their location relative to the site where the ductus arteriosus inserts. Valvular and subvalvular aortic stenosis involve narrowing at the aortic valve and below it, causing anatomical obstruction of blood flow from the LV to the aorta [8]. The severity of MV obstruction in SC is associated with a poor prognosis and earlier symptom onset. Symptoms may be non-specific, including dyspnea, cough, failure to thrive, poor feeding, lethargy, wheezing, and recurrent respiratory infections [3]. SC presents a diagnostic challenge due to its varied clinical manifestations. TTE is typically the initial imaging modality employed, providing a comprehensive assessment of LV function and significant valvular abnormalities. TTE can also help in detecting associated subaortic stenosis, supramitral ring, and CoA [1]. However, the high variability of potential defect combinations complicates diagnosis [9]. In cases of suspected or known CoA, cross-sectional imaging with cardiac magnetic resonance (CMR) or cardiac computed tomography (CCT) may be recommended to precisely define the coarctation's location, length, and diameter, guiding the selection of appropriate interventions [1]. The patient in the present case was diagnosed with partial SC through the use of TTE and CCT, which revealed the presence of a supravalvular mitral ring, sub-aortic stenosis, and CoA. In children diagnosed with this syndrome, early surgical repair is often necessary, with around 63 % of patients needing multiple surgeries [5]. Managing this group of patients is challenging due to the variable mode of presentation and the differences in the seriousness of each specific heart lesion. Recent studies have highlighted how important it is to consider the severity of the LV inflow obstruction when planning surgeries and predicting the outcomes [10]. Therefore, it is really important to have a clear and straightforward understanding of this condition in order to formulate an appropriate operative strategy. Two previous studies looking at the surgical management of Shone's syndrome suggest that it is important to address LVOT obstruction during the first surgery, and if possible, the valve should be repaired at the same time [5]. In some instances, certain intracardiac impairments were also fixed during the same surgery [7]. Herein, surgical intervention was conducted to address severe aortic isthmus stenosis, while moderate aortic and mitral stenosis treatment was deferred until the child's growth is complete. This is consistent with the literature, which indicates a high failure rate for valve replacement in children, particularly in infants under one year of age [10]. Furthermore, patient-prosthesis mismatch (PPM) may arise when a prosthetic valve's area is too small for the body surface area, as a result of somatic growth of cardiac tissue. Consequently, almost one-third of patients with CHD require interventions in adulthood mainly for bioprosthetic valve degeneration and PPM from somatic growth [5]. Given the complications of mechanical MV in children, there has been a strong focus on reconstructive strategies. Moreover, studies indicate that long-term outcomes of valve repair outperform those of replacement in individuals with isolated congenital mitral anomalies [10]. Early surgical intervention in patients with SC can lead to good outcomes, particularly before pulmonary hypertension develops. Poor surgical outcomes are associated with the severity of MV involvement and secondary pulmonary hypertension [8]. Finally, left outflow tract obstruction is initially addressed through surgical intervention, while inflow deformities are treated later. Reparative techniques should be prioritized whenever possible, with replacement considered only if repair fails.

## 4 Conclusion

This paper exemplifies a unique instance of a partial SC managed through an innovative strategy that involved staged surgical intervention for certain lesions while opting for surveillance for others, ultimately leading to improved clinical outcomes. Furthermore, early diagnosis of this entity through imaging is crucial in sustaining positive outcomes with early surgical intervention. In addition, it is imperative for emergency physicians to be aware of these lesions and the role of point-of-care ultrasound as a diagnostic tool.

## List of abbreviations

SC: Shone complex
CHD: congenital heart disease
PMV: parachute mitral valve
CoA: coarctation of the aorta
beats/min: beats per minute
mm Hg: millimetres of mercury
ECG: electrocardiogram
LV: left ventricular
TTE: transthoracic echocardiography
EF: ejection fraction
MV: mitral valve
Vmax: maximum jet velocity
PASP: pulmonary arterial systolic pressure
MDCT: multi-detector computed tomographic
LVOT: left ventricular outflow tract
CMR: cardiac magnetic resonance
CCT: cardiac computed tomography
PPM: patient-prosthesis mismatch

## Author contribution

Ayham Qatza: Writing – review & editing, Writing – original draft, Data curation.

Moumina Baroudi: Writing – review & editing, Writing – original draft.

Abdullah Dukhan: Writing – review & editing, Writing – original draft.

Ahmed Sheikh Sobeh: Writing – review & editing, Writing – original draft.

Nabeha Alibrahim: Writing – review & editing, Writing – original draft.

Saleh Takkem: Writing – review & editing, Supervision.

Ayham Qatza: Submitted the final manuscript.

All authors read and approved the final manuscript.

## Consent for publication

Written informed consent was obtained from the patient's parents/legal guardian for publication and any accompanying images. A copy of the written consent is available for review by the Editor-in-Chief of this journal on request.

## Ethical approval

Ethics clearance was not necessary since the University waives ethics approval for publication of case reports involving no patients' images, and the case report is not containing any personal information. The ethical approval is obligatory for research that involves human or animal experiments.

## Guarantor

Ayham Qatza.

## Research registration number

This case report is not a first time of reporting, new device or surgical technique. So I would not need a Research Registry Unique identifying number (UIN).

## Methods

The work has been reported in line with the SCARE criteria [11].

## Declaration of Generative AI and AI-assisted technologies in the writing process

None.

## Funding

The author(s) received no financial support for the research, authorship, and/or publication of this article.

## Conflict of interest statement

The author(s) declared no potential conflicts of interest with respect to the research, authorship, and/or publication of this article.

## Data Availability

Data sharing not applicable to this article as no datasets were generated or analyzed during the current study.
